# Changes in the Size of the Active Microbial Pool Explain Short-Term Soil Respiratory Responses to Temperature and Moisture

**DOI:** 10.3389/fmicb.2016.00524

**Published:** 2016-04-19

**Authors:** Alejandro Salazar-Villegas, Evgenia Blagodatskaya, Jeffrey S. Dukes

**Affiliations:** ^1^Department of Biological Sciences, Purdue UniversityWest Lafayette, IN, USA; ^2^Purdue Climate Change Research Center, Purdue UniversityWest Lafayette, IN, USA; ^3^Department of Soil Science of Temperate Ecosystems, University of GöttingenGöttingen, Germany; ^4^Department of Soil C and N Cycles, Institute of Physicochemical and Biological Problems in Soil Science, Russian Academy of SciencesPushchino, Russia; ^5^Department of Forestry and Natural Resources, Purdue UniversityWest Lafayette, IN, USA

**Keywords:** soil respiration, microbial dormancy, microbial biomass, substrate-induced growth response, carbon pool

## Abstract

Heterotrophic respiration contributes a substantial fraction of the carbon flux from soil to atmosphere, and responds strongly to environmental conditions. However, the mechanisms through which short-term changes in environmental conditions affect microbial respiration still remain unclear. Microorganisms cope with adverse environmental conditions by transitioning into and out of dormancy, a state in which they minimize rates of metabolism and respiration. These transitions are poorly characterized in soil and are generally omitted from decomposition models. Most current approaches to model microbial control over soil CO_2_ production relate responses to total microbial biomass (TMB) and do not differentiate between microorganisms in active and dormant physiological states. Indeed, few data for active microbial biomass (AMB) exist with which to compare model output. Here, we tested the hypothesis that differences in soil microbial respiration rates across various environmental conditions are more closely related to differences in AMB (e.g., due to activation of dormant microorganisms) than in TMB. We measured basal respiration (SBR) of soil incubated for a week at two temperatures (24 and 33°C) and two moisture levels (10 and 20% soil dry weight [SDW]), and then determined TMB, AMB, microbial specific growth rate, and the lag time before microbial growth (*t*_*lag*_) using the Substrate-Induced Growth Response (SIGR) method. As expected, SBR was more strongly correlated with AMB than with TMB. This relationship indicated that each g active biomass C contributed ~0.04 g CO_2_-C h^−1^ of SBR. TMB responded very little to short-term changes in temperature and soil moisture and did not explain differences in SBR among the treatments. Maximum specific growth rate did not respond to environmental conditions, suggesting that the dominant microbial populations remained similar. However, warmer temperatures and increased soil moisture both reduced *t*_*lag*_, indicating that favorable abiotic conditions activated soil microorganisms. We conclude that soil respiratory responses to short-term changes in environmental conditions are better explained by changes in AMB than in TMB. These results suggest that decomposition models that explicitly represent microbial carbon pools should take into account the active microbial pool, and researchers should be cautious in comparing modeled microbial pool sizes with measurements of TMB.

## Introduction

Microbial respiration responds rapidly to changing environmental conditions, strongly influencing soil carbon cycling, and its feedbacks to climate change (Allison et al., 2010; Frey et al., 2013; Wieder et al., 2013; Sulman et al., 2014). However, soil biogeochemical processes are primarily driven by only a small fraction of soil microbes—those that are physiologically active (Blagodatskaya and Kuzyakov, 2013). In general, more than 80–90% of soil microorganisms are in a dormant or inactive physiological state in which they have minimal respiratory activity (Anderson and Domsch, 1985; Lennon and Jones, 2011). These active and dormant fractions of soil microbial biomass can change in response to environmental and nutritional conditions (Van de Werf and Verstraete, 1987) but typically are not considered when analyzing microbial control over soil CO_2_ production. If they have represented microbes at all, decomposition models have most commonly represented microbial biomass as a single pool (Zhang et al., 2014; Wieder et al., 2015) without differentiating between its active and dormant fractions. This approach cannot sufficiently represent physiological processes that are important to explain soil respiratory responses to environmental conditions (Wang G. et al., 2014; He et al., 2015). Since active microbes overwhelmingly drive soil carbon processes, we investigated whether active microbial biomass (AMB) is a more accurate predictor of soil CO_2_ fluxes than total microbial biomass (TMB).

Dormancy is a common strategy in nature, used by a variety of organisms to cope with adverse environmental conditions (Dworkin and Shah, 2010; Jones and Lennon, 2010; Lennon and Jones, 2011). Although, there are different ways by which soil microorganisms become dormant (e.g., spore formation by *Scutellospora castanea* or thick-walled structure formation in filaments of *Cylindrospermum* sp.) (Jones and Lennon, 2010), in all cases there is a strong reduction of physiological activity (Lennon and Jones, 2011). In this state of reduced metabolic activity, microorganisms have almost no influence on biogeochemical processes such as soil CO_2_ production. However, dormant microorganisms can be activated when adverse environmental and nutritional conditions become favorable (Jones and Lennon, 2010; Placella et al., 2012; Aanderud et al., 2015). These transitions between active and dormant physiological states may play an important role in large-scale processes such as global carbon cycling (He et al., 2015).

Differentiation between the pools of active and dormant microbial biomass could provide important opportunities to better understand responses of soil CO_2_ efflux to environmental factors, e.g., temperature and soil moisture. In regions such as the Mediterranean, rainfall-induced activation of dormant microorganisms generates soil CO_2_ pulses that approach the annual net carbon exchange of other terrestrial ecosystems (Xu et al., 2004; Placella et al., 2012). Conversely, when the frequency of rainfall events in a region causes drying-rewetting stress on soil microbial communities, SBR can decrease even if TMB increases (Fierer and Schimel, 2002). This could be a consequence of smaller fractions of physiologically active microorganisms in environmentally stressed soils (Fierer and Schimel, 2002) or of shifts toward microbial communities with higher carbon use efficiency. In addition to soil moisture conditions, microbial respiration, growth, and activity can respond strongly to temperature (Pietikåinen et al., 2005; Steinweg et al., 2012; Suseela et al., 2012; Hagerty et al., 2014). Like soil moisture, warming can increase SBR without affecting TMB (Hagerty et al., 2014), potentially via activation of dormant microorganisms. Thus, TMB does not always respond to environmental changes (Holmes and Zak, 1994; Blagodatskaya et al., 2010) and it remains unclear whether its responses (e.g., TMB decrease under water or nutrient limitation) are proportional to those of AMB.

Current approaches to modeling microbial control over soil CO_2_ production mainly consider changes in TMB (Wieder et al., 2015) and essentially ignore changes in the pools of active and dormant microbial biomass (e.g., due to activation of dormant microorganisms; Wang G. et al., 2014). This may be because the current microbial databases available to modelers only represent TMB (Serna-Chavez et al., 2013; Xu et al., 2013) and do not distinguish its active and dormant fractions.

To quantify the importance of active and dormant microbial pools in explaining soil respiratory responses to abiotic factors, we incubated soil at different temperature and moisture levels for a week and subsequently analyzed the correlation of SBR with TMB and AMB. Since, (1) only active microorganisms are able to drive soil biogeochemical processes and (2) abiotic factors can cause microorganism to be activated or inactivated without necessarily altering TMB, we hypothesized that soil respiratory responses to changing environmental conditions would be better explained by changes in AMB than in TMB. We used a kinetic approach based on the Substrate Induced Growth Response (SIGR) technique (Panikov and Sizova, 1996; Blagodatsky et al., 2000; Wutzler et al., 2012) to measure TMB and AMB, as well as other microbial parameters (i.e., microbial specific growth rate and the length of the lag-time before exponential growth starts in response to substrate inputs *t*_*lag*_) that help shed light on the mechanisms by which microbes influence soil CO_2_ production.

## Materials and methods

### Soil sampling and preparation

We collected three soil cores separated by 10 m (linear transect) from the top 0–15 cm layer (using a soil core sampler and slide hammer; AMS, Inc.) in a deciduous forest at Purdue University's Ross Biological Reserve, Indiana, USA (40° 24′46″ N, 87° 03′48″ W), in May 2014. The soil is classified as (2–6% slope) Russell (Alfisol) silt loam (USDA, 2014) and has a pH of 6.97. The mean annual temperature and mean annual precipitation at this site are 11.4°C and 953 mm, respectively (USDA, 2014).

Immediately after sampling, we transported the soil cores to the laboratory (<1 h, at ambient temperatures), prepared a composite sample by sieving the soil through a 2 mm mesh, and adjusted the soil moisture content to 10% (moisture deficit) and 20% (optimal soil moisture) of soil dry weight (SDW). Finally, we placed 25 g of soil (dry weight) in 0.26 L septum-capped glass jars (microcosms) and stored them at 24 and 33°C for 1 week (see Section Experimental Setup below).

### Experimental setup

After soil samples had been incubated for 1 week at 24 and 33°C, (hereafter unheated and heated) and 10 and 20% SDW soil moisture (hereafter dry and wet) conditions, we indirectly measured TMB and AMB (and other microbial parameters) using soil respiratory responses (SIGR) to glucose and mineral nutrient inputs (Panikov and Sizova, 1996). To induce these responses, we homogeneously spread a solution (1 mL per jar) containing 10 mg glucose, 1.90 mg (NH_4_) SO_4_, 2.25 mg K_2_HPO_4_, and 3.62 mg MgSO_4_ per gram of soil (Blagodatskaya et al., 2014) onto the soil in each jar. The addition of 1 mL solution per sample increased the soil moisture in dry and wet soils from 10 to 20%, during the incubation period, to 14 (still below optimum) and 24% (still within optimal moisture range) SDW during the substrate-induced growth period. We defined the temperature and soil moisture treatments based on ranges of environmental conditions commonly experienced by soil microbial communities in the study area (Goldberg, 2015; ICLIMATE, 2015). Within these ranges, warming and increased soil moisture generally increase soil respiration rates (Li et al., 2008; Yu et al., 2011; Suseela et al., 2012). To create the warming treatment and maintain constant soil moisture we stored the jars in an Environmental Growth Chamber (M18SI) during the incubation period, kept them closed, and confirmed that there were no changes in soil weight (i.e., due to water losses). We measured the CO_2_ concentration in each microcosm's headspace every 0.5 for 4 h before adding the solution, and every 0.5 for 19 h (exponential growth phase) afterwards. We measured CO_2_ concentration by withdrawing 5 mL of gas from the microcosm headspace using a syringe and injecting it into an infrared gas analyzer (EGM-4, PP Systems, Amesbury, Massachusetts, USA). To avoid negative pressure, we opened the jars at the end of each measurement (i.e., after measuring *C*_1_), aerated the headspace until the CO_2_ concentration in the microcosms equilibrated with ambient air, closed the jars, and withdrew the initial gas sample (i.e., *C*_0_) of the following measurement.

We calculated soil respiration rates as in Speratti and Whalen (2008):

$$\begin{array}{l} R_{s}\left(t\right)=\frac{V\left(C_{1}-C_{0}\right)}{Wt} \end{array}$$

Where *R*_*s*_*(t)* is soil respiration rate at time *t* (in mg C g^−1^ h^−1^), *V* is the volume of the microcosm headspace, *C*_1_–* C*_0_ is CO_2_ concentration change in mg L^−1^, *W* is the dry mass of the soil (i.e., 25 g), and *t* is the time between the first (*C*_0_) and the second (*C*_1_) CO_2_ concentration measurements (i.e., 0.5 h).

### Kinetic respiration analysis

To estimate TMB, AMB, microbial specific growth rate, and *t*_*lag*_, we used the model proposed by Panikov and Sizova (1996) (see also Wutzler et al., 2012):

$$\begin{array}{l} R_{s}\left(t\right)=R_{u}+R_{c}*\exp\left(\mu t\right) \end{array}$$

Where *R*_*u*_ is initial respiration rate uncoupled from growth, *R*_*c*_ is initial respiration rate coupled with growth, and μ is microbial specific growth rate (an intrinsic feature of microbial species). μ is defined as the slope of the growth curve at its inflection point when nutritional and environmental conditions are optimum (Pirt, 1982; Zwietering et al., 1990). At non-optimum conditions, μ is not maximal and reflects environmental constraints of growth rather than any intrinsic feature of the dominant microbial population (Pirt, 1982). It is important to note that soil moisture in the dry treatment (10% SDW) was below optimum (generally 20–40% sdw; Ilstedt et al., 2000) during the period of substrate-induced growth, so μ reflects growth limited by moisture. However, since excessive amounts of substrate were added and homogeneously spread in the soil, the main assumption of exponential growth in an excess of substrate was valid in all treatments. Violating assumptions on optimal growth conditions related to temperature and moisture affect estimation of maximal specific growth rates (μ_*max*_). To avoid violating this assumption, only specific growth rates (μ) were calculated for different treatments. AMB was estimated based on substrate-induced exponential growth curves. Differences in exponential curves reflect differences in AMB, caused by the treatments, at the time point immediately before substrate addition.

We fitted the model parameters to measured soil respiration rates *(R*_*s*_*)* during the lag and exponential phases that followed substrate amendment. We omitted the first 3 h of measurements from the analysis to exclude the transient effects of mixing and preparing the soil on soil respiration rates (Wutzler et al., 2012).

We estimated TMB as:

$$\begin{array}{l} TMB=\frac{R_{c}\lambda Y_{CO_{2}}}{r_{0}\mu } \end{array}$$

Where λ is a basic stoichiometric constant assumed to equal 0.9, which represents the ratio between productive (i.e., respiration that is coupled with ATP generation and cell growth) and total respiration under excess of substrate (Akimenko et al., 1983; Panikov and Sizova, 1996); *Y*_*CO*2_ is another constant, the biomass yield per unit of respired CO_2_, assumed to equal 1.5 for soil heterotrophs (Payne, 1970; Blagodatsky et al., 2000); and *r*_0_ is the *fraction of AMB*, given by:

$$\begin{array}{l} r_{0}=\frac{R_{c}\left(1-\lambda \right)}{R_{u}+R_{c}\left(1-\lambda \right)} \end{array}$$

Where the numerator accounts for the maintenance respiration of growing (i.e., active) microbial biomass, and the denominator accounts for total maintenance respiration.

Finally, we estimated AMB as:

$$\begin{array}{l} AMB=TMB*r_{0} \end{array}$$

To better understand the mechanisms by which soil microbes control soil carbon dynamics, we also calculated *t*_*lag*_ as in Blagodatskaya et al. (2014).

tlag=ln(RuRc)μ

Note that these estimates of AMB, TMB, and *r*_0_ are for the time period immediately preceding substrate amendment, despite being derived from measurements that follow substrate amendment.

### Statistical analysis and curve fitting

We estimated SBR as the mean of the eight soil respiration measurements taken in the 4 h prior to substrate amendment. For the CO_2_ evolution curve fitting we used the non-linear least square (nls) function in R (version 3.1.1). We used R^2^ as a measure of goodness of fit.

To estimate the significance of the differences in SBR and microbial parameters between treatments, we applied a two-way ANOVA using the aov() function in R (version 3.1.1). When significant differences were found, we conducted post-hoc pairwise comparisons using the TukeyHSD function (these results are shown in the Supplementary Material section). To meet assumptions regarding the normality of residuals and homogeneity of variances we log_10_-transformed AMB for statistical analyses, but for clarity we present the untransformed data in the text and figures. All values are means of three replicates per treatment.

We calculated the percentage of the variance explained by each independent factor by dividing the sum of squares of each factor (from the ANOVAs mentioned above) by the total sum of squares (examples of calculations based on sums of squares and statistics are in Supplementary Table 1).

## Results

**Figure 1:**
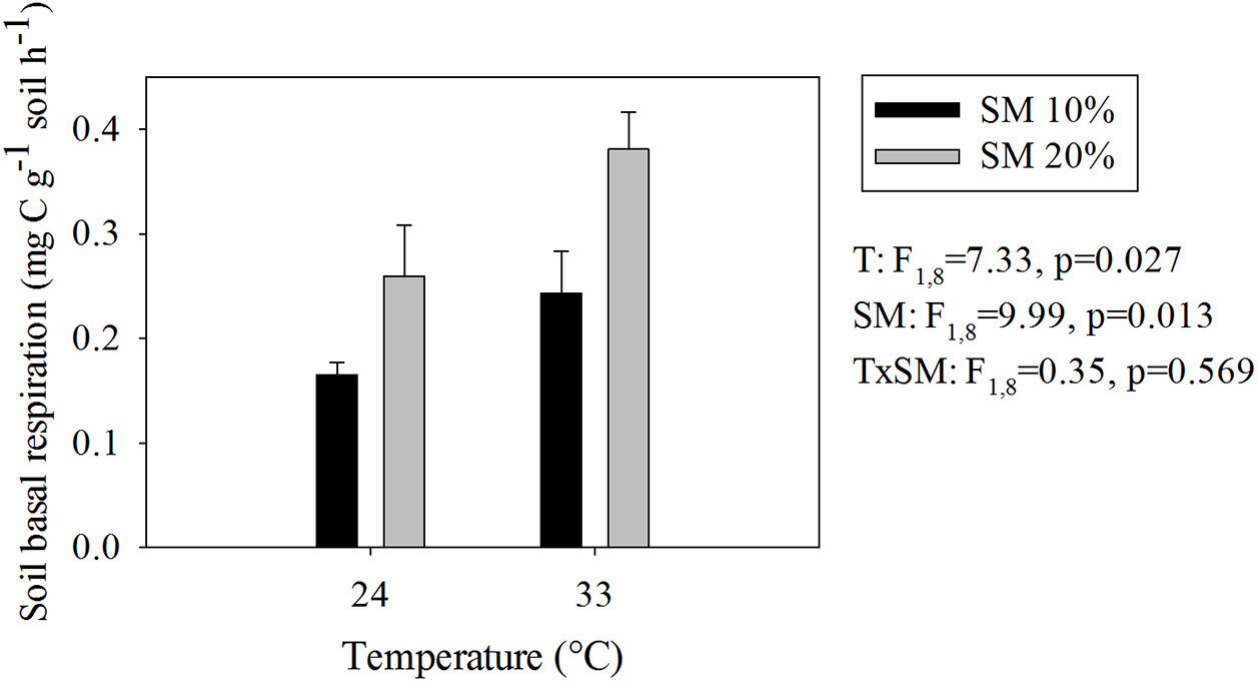
**Soil basal respiration rates at different temperature and soil moisture conditions**. Values represent means ± SE. SM, Soil moisture.

As we expected, warmer and wetter conditions both significantly increased SBR (Figure 1). The highest SBR occurred in heated, wet soils, whereas the unheated, dry soils had the lowest (Figure 1) Soil CO_2_ production in heated, wet soils was 2.4 times higher than in unheated, dry soils.

**Figure 2:**
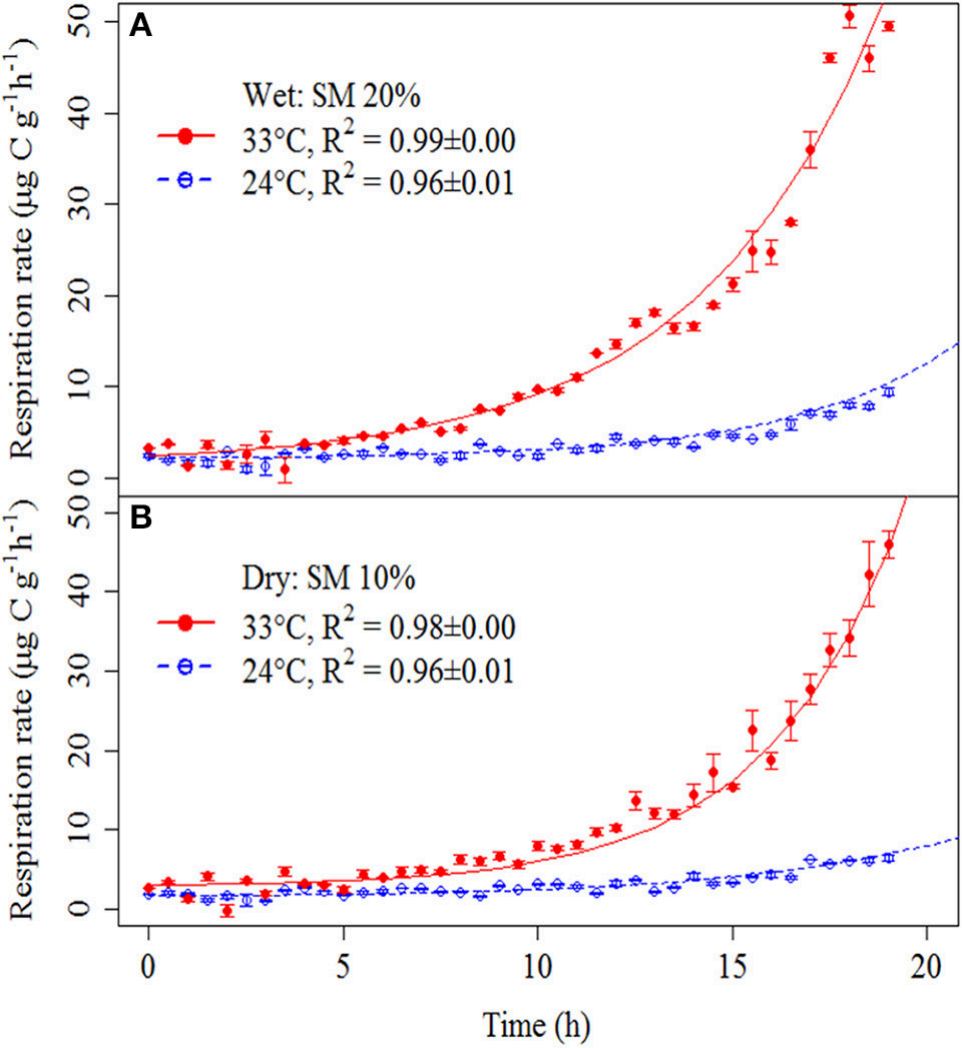
**Soil respiration rates after addition of a glucose and nutrient solution in wet (A) and dry (B) soils at different temperatures (heated soils, red solid circles/solid line; and unheated soils, open blue circles/dashed line)**. Symbols represent means ± SE. Lines were obtained by fitting the model parameters to measured soil respiration rates (see Materials and Methods Section). Fitted parameters are in Supplementary Table 2. Fitted lines are based on mean parameters values for each treatment. *R*^2^-values were calculated based on linearized model (Supplementary Figure 1) but exponential curves are shown to illustrate the exponential nature of the SIGR.

Soil respiration curves showed clear responses to substrate addition, with particularly marked differences in response between heated and unheated treatments (Figure 2).

**Figure 3:**
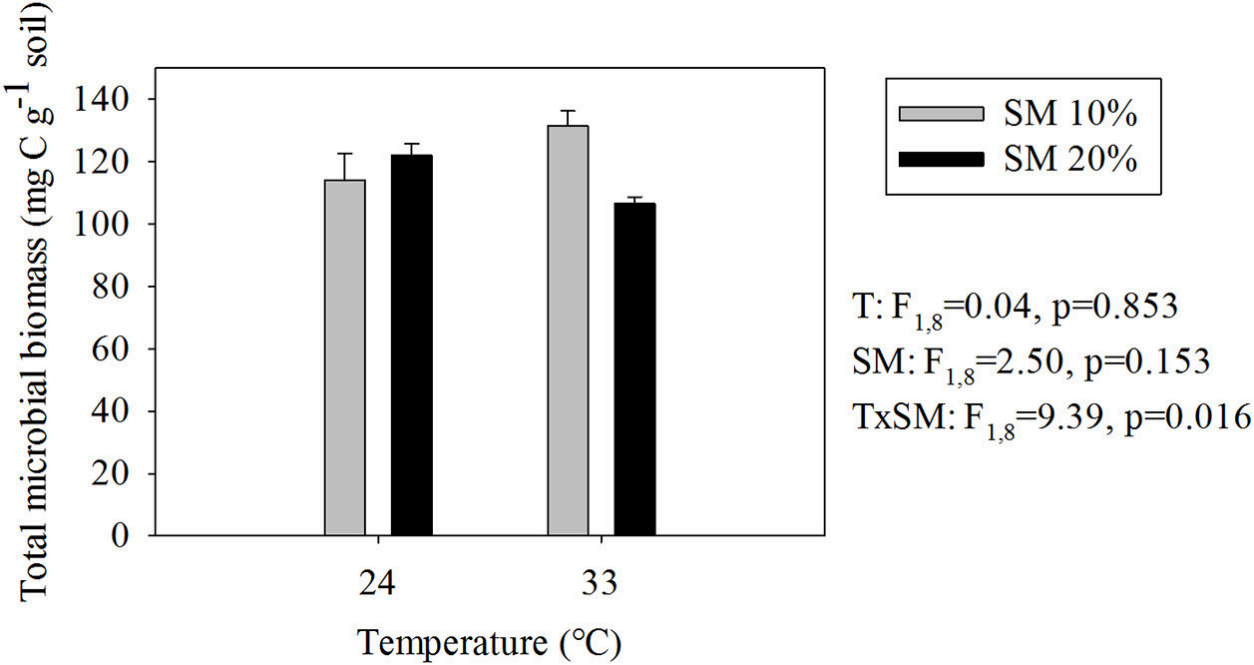
**Estimated total microbial biomass (means ± SE; *n* = 12) at different temperature and soil moisture conditions**. SM, Soil moisture.

Responses of TMB to warming and soil moisture differed from those of SBR (compare Figures 1, 3). The highest TMB occurred in heated, dry soils whereas the lowest occurred in heated, wet soils (i.e., 19% decrease in TMB due to a soil moisture increase in heated soils; Figure 3). Soil moisture level did not significantly affect TMB of unheated soils.

**Figure 4:**
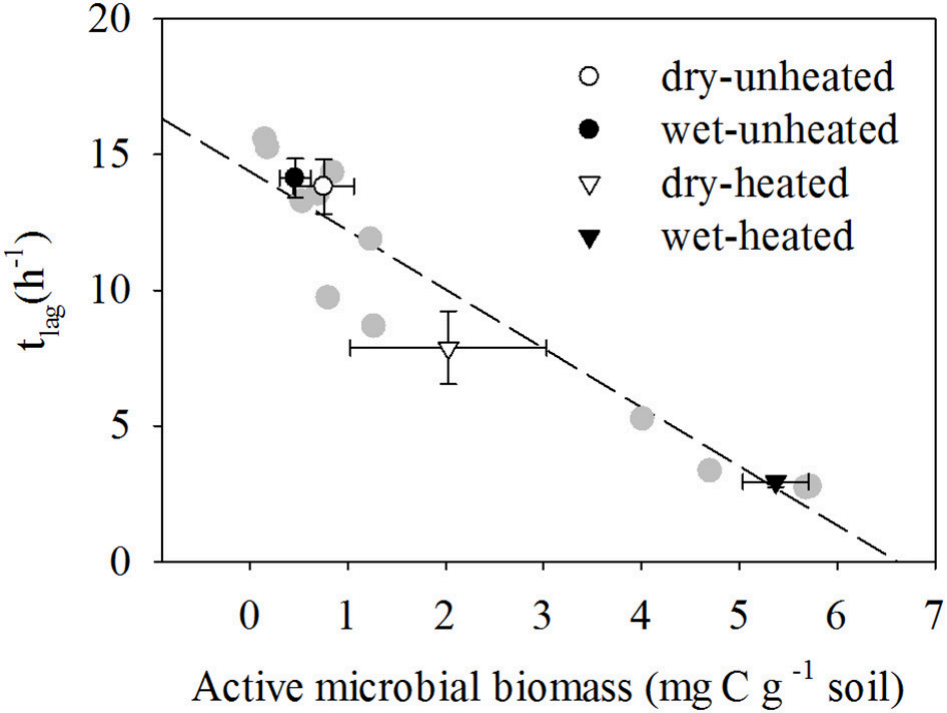
**The relationship between *t*_*lag*_ and active microbial biomass at different temperature and soil moisture conditions (means ± SE shown in black and white symbols; *n* = 12)**. SM, Soil moisture. Individual replicates shown in gray, and two replicate observations at 33°C and 20% SM overlap.

In contrast to TMB, the responses of AMB to temperature and soil moisture were similar to those of SBR (Figure 4). As with SBR, warming increased AMB and the greatest AMB occurred in heated, wet soils. Unheated soils had the least AMB. While moisture did not affect AMB in the unheated soils, it increased AMB by 250% in heated soils.

AMB responses to warming and soil moisture were strongly negatively correlated with *t*_*lag*_ [*R*^2^ = 0.936, *F*_(1, 11)_ = 145.51, *P* < 0.001; Figure 4]. Warming reduced *t*_*lag*_ by 43% in dry treatments and by 79% in wet soils. In contrast, μ did not respond to temperature and soil moisture Supplementary Tables 9,10 and was less correlated with AMB [*R*^2^ = 0.14, *F*_(1, 11)_ = 1.6, *P* = 0.235].

**Figure 5:**
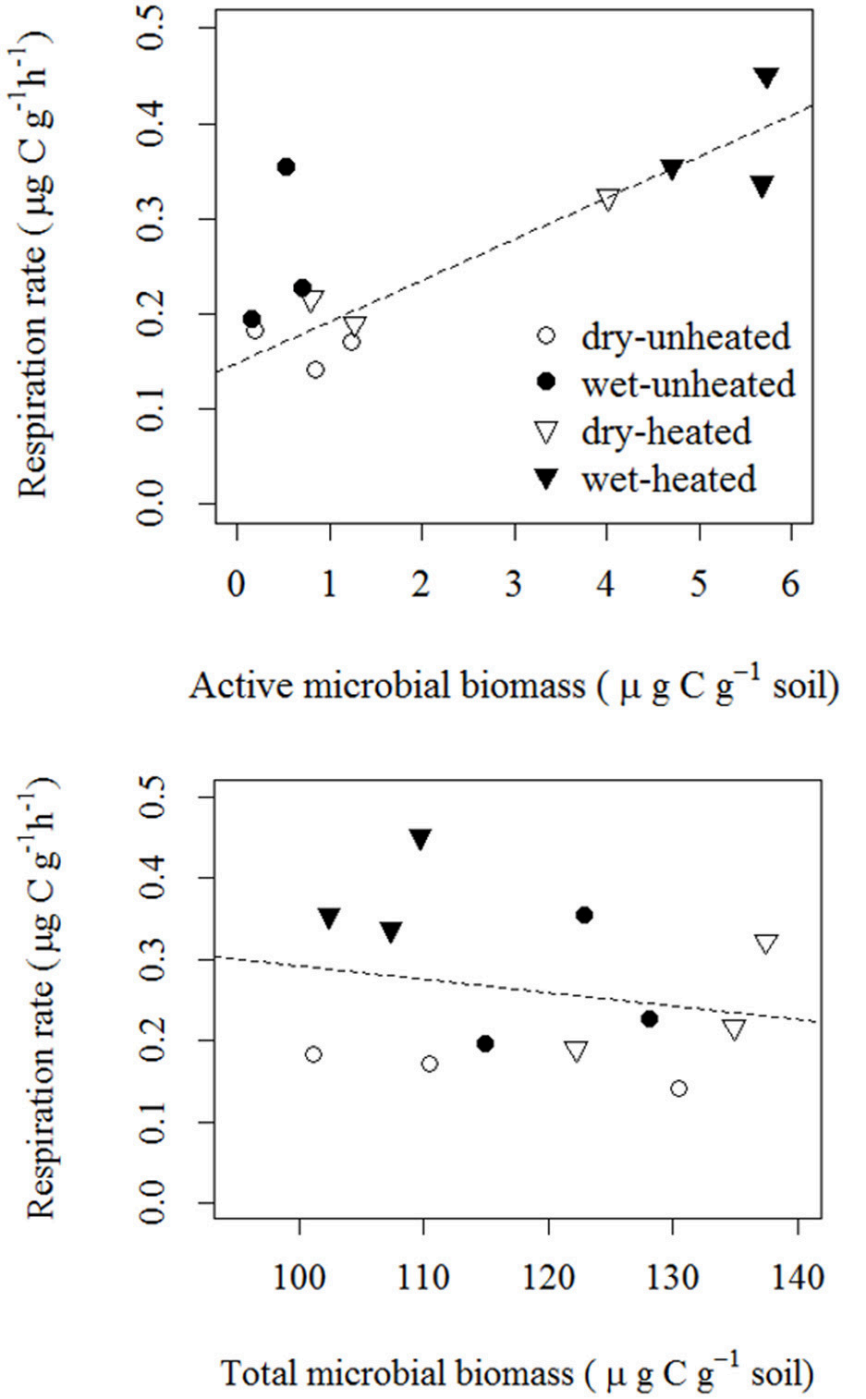
**Correlations of soil basal respiration with active microbial biomass [top; *F*_(1, 11)_ = 15.97, *p* = 0.003 *R*^2^ = 0.62; statistics were calculated using log_10_-transformed AMB] and total microbial biomass [bottom; *F*_(1, 11)_ = 0.47, *p* = 0.509, *R*^2^ = 0.04**].

As we expected, soil respiration rates across the temperature and soil moisture treatments were strongly correlated with AMB, but not with TMB (Figure 5). The correlation between AMB and SBR was positive and indicated an approximate increase of 0.04 μg CO_2_-C g^−1^ soil h^−1^ per μg active biomass C g^−1^ soil.

**Figure 6:**
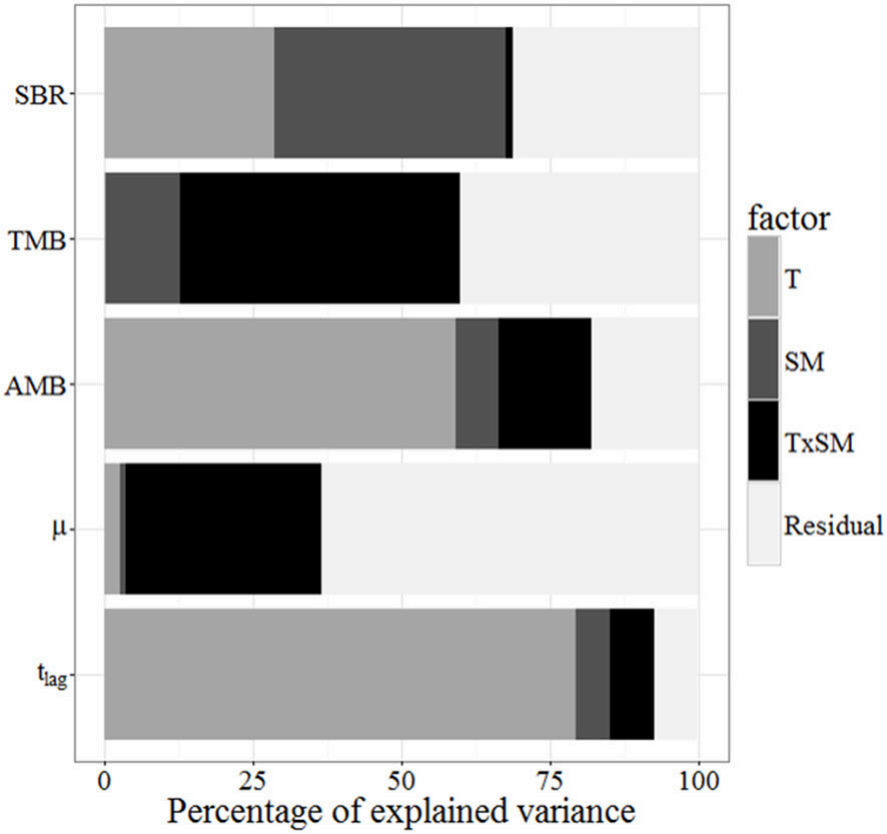
**Contributions of temperature, soil moisture, and their interactions to the variance of soil basal respiration, total microbial biomass, active microbial biomass, μ, and *t*_*lag*_, measured as described in Section Statistical Analysis and Curve Fitting**. T, Temperature; SM, Soil moisture.

Temperature typically explained more of the variation in soil microbial parameters (especially AMB and *t*_*lag*_) than did soil moisture (Figure 6). However, both environmental factors were important in explaining variation in SBR, and the two together accounted for 70% of this variation. This analysis accounts for the direct effects of temperature and moisture on SBR (e.g., 29% of variance explained by temperature) and microbial parameters (e.g., 59% of variance in AMB explained by temperature). In contrast, the relationships between SBR and microbial biomass (Figure 5) suggest that microbial activation was the biological mechanism through which these abiotic factors influenced soil respiration rates (e.g., 62% of the variance in SBR was explained by environmentally—mainly temperature—driven changes in the pool of AMB).

## Discussion

Researchers long ago established that soil microbial respiration responds to changes in environmental conditions (Birch, 1958), but only recently have soil microbial processes been explicitly incorporated into soil carbon models (Fujita et al., 2014; Zhang et al., 2014; Wieder et al., 2015). The most common approach has been to represent microbial biomass as a single pool, without differentiating between microorganisms in active and dormant physiological states. Here, the observed increases in soil CO_2_ efflux in response to warming and increased soil moisture were explained by an increase in the active fraction of the soil microbial community rather than by any increase in the size of the total community (i.e., TMB). As we predicted, SBR was more strongly correlated with AMB than with TMB. An increase in AMB after warming and wetting with no significant change in TMB suggests a quick shift of dormant microorganisms to an active physiological state, which corresponded to the increase in respiration. Given that the specific growth rate is an intrinsic feature of the microbial population (Panikov, 1995; Rousk and Bååth, 2007), the insensitivity of μ-values to soil wetting and warming indicates that this microbial activation did not dramatically change the functional structure of the microbial community (i.e., the dominant population growing on added substrate). Moreover, the stronger correlation of *t*_*lag*_ with AMB than with μ supports an observation that lag time is regulated primarily by activity state rather than by maximal specific growth rate, even though both parameters are used in calculation of *t*_*lag*_ (Blagodatskaya et al., 2014). Overall, these results are consistent with observations from other studies in which soil respiration responses to warming (Hagerty et al., 2014) and soil moisture (Fierer and Schimel, 2002; Butterly et al., 2009; Placella et al., 2012) were not explained by changes in TMB but by changes in the physiology of soil microbial communities, such as resuscitation of physiologically clustered microbial groups (Placella et al., 2012; Aanderud et al., 2015; Barnard et al., 2015).

Direct effects of temperature and moisture on SBR can differ in magnitude from those on specific respiration-related microbial processes (e.g., activation of dormant biomass). The combined analysis of the proportion of variances explained by abiotic factors and the relationships between SBR and total and active MB suggests that SBR responses to temperature are strongly linked to changes in AMB. The activation of dormant microorganisms under warmed conditions raises the question of how and why environmental factors trigger activation of different microbial taxa. While we cannot directly address these questions here, we speculate that the temperature of heated soils may have been closer to the optimum for microbial processes than that of unheated soils. On the other hand, moisture explained a larger portion of the variation in SBR than in AMB. This could be explained in part by the fact that AMB, but not SBR, was insensitive to moisture under unheated conditions. This suggests that the importance of microbial physiology for explaining soil carbon processes could vary across moisture (Manzoni et al., 2016) and temperature gradients. The proportions of the variances explained by temperature and moisture also differed between SBR and TMB. This further supports the lack of a relationship between TMB and SBR, at least at the temporal scales relevant for this study.

Different responses of TMB and AMB to environmental conditions may be explained by recycling of soil nutrients. The 1-week incubation at optimum warming and soil moisture conditions (i.e., the warmer and wetter soils) decreased TMB but increased AMB, suggesting that part of the dead biomass was re-utilized by the active microbial fraction. By recycling energy and nutrients from dead biomass, microorganisms in the more favorable conditions likely remained in an active state, which reduced total biomass, but maximized active biomass and led to the highest fraction of active biomass (5.0 ± 0.2% of TMB) in the warm and wet treatment. Rapid declines in TMB after re-moistening of pre-conditioned soil have been detected under laboratory conditions without substrate addition (Butterly et al., 2009; Blagodatskaya et al., 2011; Tian et al., 2015). Our study revealed that such a decline in TMB is not necessarily accompanied by a decrease in AMB, suggesting a possible mechanism for maintaining activity under unfavorable conditions (e.g., starvation). Similar results have been observed in soil from an old-field experiment in Wagga Wagga, NSW, Australia, where single drying-rewetting events strongly decreased microbial biomass C and P, but increased microbial activity (Butterly et al., 2009). Decreases in TMB accompanied by increases in AMB have also been observed at the seasonal scale: TMB and AMB decreased and increased, respectively, from January to June in fallow and winter wheat soils from an experimental field in Hélécine, Belgium (Van de Werf and Verstraete, 1987). Taken together, these observations suggest that, at both short-term (i.e., few days) and seasonal scales, TMB and AMB can have different and even opposite responses to environmental conditions.

Another explanation of the phenomena observed in this study could be that warm and moist conditions quickly activated the grazing of microbial biomass by protozoans (Pomeroy, 1974), rapidly decreasing microbial biomass and enabling rapid nutrient recycling by protozoan grazers (Bonkowski et al., 2000). Nutrients released by protozoans could have facilitated microbial turnover, increasing the active fraction of the microbial community (Blagodatskaya et al., 2014). Conversely, drier conditions may have prevented such fast self-digesting by reducing the activity of protozoa, which are very sensitive to drought (Geisen et al., 2014). We conclude that the mechanisms controlling soil nutrient recycling (e.g., reutilization of dead biomass or belowground grazing) drive the different responses of TMB and AMB to environmental conditions.

At the seasonal scale, variations in soil respiration rates are generally much larger than variations in TMB. Soil respiration rates are faster in the growing season than in winter in a variety of ecosystems (Grogan and Chapin, 1999; Suseela et al., 2012; Suseela and Dukes, 2013; Keidel et al., 2014; Reynolds et al., 2014; Wang Y. et al., 2014;). Typically, these variations closely follow changes in temperature and soil moisture (Grogan and Chapin, 1999; Suseela et al., 2012; Suseela and Dukes, 2013). Across seasons, TMB generally varies less than soil heterotrophic respiration (Holmes and Zak, 1994; Gunapala and Scow, 1998; Blume et al., 2002; but see Devi and Yadava, 2006). Our results suggest that this difference could be explained in part by seasonal changes in the pools of active/dormant microbial biomass. However, this hypothesis remains untested.

At the global scale, net changes in the size of active and dormant microbial biomass pools (e.g., due to climate change) could strongly affect biogeochemical processes such as carbon cycling. Transitions between active and dormant physiological states have been incorporated into some dynamic microbial community models to simulate community responses to environmental changes, such as drying-rewetting cycles (Konopka, 1999; Bär et al., 2002; Stolpovsky et al., 2011). However, few attempts have been made to include these transitions in larger, ecosystem-scale models (Manzoni et al., 2014; Wang G. et al., 2014; He et al., 2015; Tang and Riley, 2015). Although other microbial-based models only consider TMB (Wieder et al., 2015), microbial biomass in these models is capable of growing and readily responding to substrate inputs. Thus, in practice, these models actually simulate the active fraction of TMB (although they ignore changes in the AMB pool due to active ↔ dormant biomass transitions). Our results suggest that it would be more appropriate to validate/calibrate these models with a microbial database that includes AMB or the active fraction of TMB than with databases that only consider TMB (Serna-Chavez et al., 2013; Xu et al., 2013). Currently, though, there are not enough empirical data to create such a database, and our understanding of spatial and temporal patterns of AMB remains rudimentary. Some models have been validated based on TMB data (Wang G. et al., 2014; Buchkowski et al., 2015; He et al., 2015). In agreement with our findings, TMB has been found to be far less responsive to external factors than predicted by models, and a poor predictor of soil respiration rates (Buchkowski et al., 2015). Attempts to validate models that explicitly represent active/dormant pools using TMB data have resulted in estimates of large fractions of active biomass (70–90%, Wang G. et al., 2014) that seem unrealistic when compared with our findings and with most current reports (generally <10–20%, Lennon and Jones, 2011), or that produce simulated relationships that are not supported by our findings (e.g., more variation of AMB explained by moisture than by temperature, He et al., 2015). Overall, incorporation of microbial dormancy in ecosystem models has facilitated model development and validation (Wang et al., 2015) produced more accurate predictions of soil heterotrophic respiration and microbial biomass (He et al., 2015), and led to predictions of weaker carbon-climate feedbacks than those given by microbial models that do not represent active/dormant biomass pools (Tang and Riley, 2015). Taken together, this suggests that incorporation of dormancy in ecosystem models influences predictions of future carbon-climate feedbacks and leads to a more realistic (and still mathematically synthesizable, computationally plausible, and experimentally testable) representation of microbial influences on soil carbon cycling. This also highlights the need for experimental work that tests these hypotheses at large spatio-temporal scales.

We know of only three previous studies that have investigated the relationship between AMB and microbial respiration in soils (Alvarez et al., 1998; Aanderud et al., 2015; Barnard et al., 2015), and only one of these studies (Barnard et al., 2015) was designed to examine relationships among environmental conditions, AMB, TMB, and soil respiration. The paucity of previous studies limits our ability to extrapolate from the AMB-respiration relationship that we observed to AMB-respiration relationships in other ecosystems and environmental conditions. In our study, each gram of C in AMB was associated with the emission of ~0.04 g CO_2_-C h^−1^. In a pasture topsoil (0–15 cm) from Pergamino, Argentina, this relationship varied from 0.01 to 0.18 g CO_2_-C h^−1^ per g active biomass C, depending on the availability of labile C (Alvarez et al., 1998). This variation was explained by changes in the composition of the active microbial pool, or by changes in the physiology of the extant soil microbial community (e.g., prevalence of aged cells with lower CO_2_ production per unit of biomass in soils with scarce or inaccessible labile substrates). The CO_2_ released from 15-cm deep cores of California grassland soils in the first 2 h after a rewetting event ranged from 8 to 33 mmol m^−2^, depending on the precipitation pattern preceding the rewetting event. This peak in soil respiration was strongly linked (*R* = 0.83, *p* < 0.001) to increases in the abundance of potentially active bacteria (Barnard et al., 2015). Similarly, in agricultural, grassland, and forest soils from southwestern Michigan, USA, the resuscitation of “rare biosphere” (defined as soil bacterial taxa that were not detectable in dry soils but became detectable after a rewetting event) was associated with 5–20 fold increases in net production of soil CO_2_ (Aanderud et al., 2015). Other studies have quantified fractions of AMB across a variety of systems. Estimates range from 4 to 49%, depending on season, land use, and soil depth (Van de Werf and Verstraete, 1987), 0.02 to 24.2% depending on soil age (Khomutova et al., 2004), and 0.24 to 0.32% depending on root presence (Blagodatskaya et al., 2014). However, these studies did not explicitly quantify the relationship between AMB and soil carbon flux, which limits our ability to conduct a broader analysis of the AMB-respiration relationship.

Another factor to consider when comparing observations of AMB from different studies is the diverse array of methods used to make these estimates (Blagodatskaya and Kuzyakov, 2013; Yakushev, 2015). In contrast, most TMB data have been collected using the same method: chloroform fumigation (Serna-Chavez et al., 2013; Xu et al., 2013). The different techniques used to estimate AMB have not been directly compared with one another, but typically produce AMB estimates of similar magnitudes (Blagodatskaya and Kuzyakov, 2013). The kinetic approach that we used in this study has important advantages, including that it can be used to quantify microbial responses to the environment at the community level, and that the outcomes are mathematically compatible with the type of data used to parameterize/validate models (e.g., size of carbon pools, units of carbon released per unit of AMB, etc). Other methods can give information that improves our understanding of soil microbial ecology and biochemistry, but that currently cannot be incorporated into models due to mathematical and/or computational limitations (e.g., use of molecular techniques to study the responses of specific microbial phylogenetic groups to environmental conditions). One of the caveats of the SIGR method is that it relies on assumptions (see Section Kinetic Respiration Analysis) that, despite having been exhaustively tested in many systems (Payne, 1970; Akimenko et al., 1983; Panikov and Sizova, 1996; Blagodatsky et al., 2000; Wutzler et al., 2012), may not be true in all cases. Another characteristic of this method is that it simplifies the continuous gradient between active, potentially active, and dormant biomass (Blagodatskaya and Kuzyakov, 2013) into two discrete pools: active and dormant biomass. Given the marked differences between the metabolism of microorganisms in active and dormant physiological states, and the ways they interact with their environment (Lennon and Jones, 2011), this seems a fair simplification, and a useful one for modeling purposes.

While we observed a strong response of AMB to temperature (e.g., Figure 6), and a strong link between AMB and soil respiration (e.g., Figure 5), the overall importance (and dependence on other factors, e.g., water-resource availability interactions) of changes in active/dormant microbial pools for soil processes at large spatio-temporal scales still remains uncertain. It is not clear, for example, whether climatic changes predicted for this century (e.g., warming and increased precipitation variability) could cause a net increase in global AMB by (net) activating some fraction of dormant soil microorganisms. If this were to occur, predictions of future soil CO_2_ production based on TMB only (i.e., without considering active/dormant transitions) could underestimate future soil CO_2_ emissions.

We conclude that soil respiratory responses to short-term changes in environmental conditions are better explained by changes in the active fraction of the soil microbial pool than by changes in TMB. Based on these results, we suggest that decomposition models that explicitly represent microbial processes should take into account the active microbial pool, and recommend researchers be cautious when comparing modeled microbial pool sizes with measurements of microbial biomass.

## Author contributions

AS substantially contributed to the conception, design, and critical review of the work, as well as to the acquisition, analysis, and interpretation of the data. EB substantially contributed to the analysis and interpretation of the data, as well to the critical review of the work. JD substantially contributed to the conception, design, and critical review of the work, as well as to the analysis, and interpretation of the data.

### Conflict of interest statement

The authors declare that the research was conducted in the absence of any commercial or financial relationships that could be construed as a potential conflict of interest.

## Abbreviations

AMB: active microbial biomass
SBR: soil basal respiration
SDW: soil dry weight
SIGR: Substrate-induced growth response
*t*_*lag*_: lag time before microbial growth
TMB: total microbial biomass
*μ*: microbial specific growth rate.

## References

[B1] Aanderud Z. Jones S. Fierer N. Lennon J. T. (2015). Resuscitation of the rare biosphere contributes to pulses of ecosystem activity. Front. Microbiol. 6:24. 10.3389/fmicb.2015.00024 25688238 PMC4311709

[B2] Akimenko V. Trutko S. Medentsev A. Korobov V. P. (1983). Distribution of cyanide-resistant respiration among yeasts and bacteria and its relation to oversynthesis of metabolites. Arch. Microbiol. 136, 234–241. 10.1007/BF00409851

[B3] Allison S. D. Wallenstein M. D. Bradford M. A. (2010). Soil-carbon response to warming dependent on microbial physiology. Nat. Geosci. 3, 336–340. 10.1038/ngeo846

[B4] Alvarez C. Alvarez R. Grigera M. Lavado R. (1998). Associations between organic matter fractions and the active soil microbial biomass. Soil Biol. Biochem. 30, 767–773. 10.1016/S0038-0717(97)00168-5

[B5] Anderson T.-H. Domsch K. (1985). Determination of ecophysiological maintenance carbon requirements of soil microorganisms in a dormant state. Biol. Fertil. Soils 1, 81–89. 10.1007/BF00255134

[B6] Bär M. Hardenberg J. Meron E. Provenzale A. (2002). Modelling the survival of bacteria in drylands: the advantage of being dormant. Proc. R. Soc. Lond. Series B Biol. Sci. 269, 937–942. 10.1098/rspb.2002.1958 12028777 PMC1690970

[B7] Barnard R. L. Osborne C. A. Firestone M. K. (2015). Changing precipitation pattern alters soil microbial community response to wet-up under a Mediterranean-type climate. ISME J. 9, 946–957. 10.1038/ismej.2014.192 25314319 PMC4817701

[B8] Birch H. (1958). The effect of soil drying on humus decomposition and nitrogen availability. Plant Soil 10, 9–31. 10.1007/BF01343734

[B9] Blagodatskaya E. Kuzyakov Y. (2013). Active microorganisms in soil: critical review of estimation criteria and approaches. Soil Biol. Biochem. 67, 192–211. 10.1016/j.soilbio.2013.08.024

[B10] Blagodatskaya E. Blagodatsky S. Anderson T.-H. Kuzyakov Y. (2014). Microbial growth and carbon use efficiency in the rhizosphere and root-free soil. PLoS ONE 9:e93282. 10.1371/journal.pone.0093282 24722409 PMC3982954

[B11] Blagodatskaya E. Blagodatsky S. Dorodnikov M. Kuzyakov Y. (2010). Elevated atmospheric CO2 increases microbial growth rates in soil: results of three CO2 enrichment experiments. Glob. Change Biol. 16, 836–848. 10.1111/j.1365-2486.2009.02006.x

[B12] Blagodatskaya E. Yuyukina T. Blagodatsky S. Kuzyakov Y. (2011). Turnover of soil organic matter and of microbial biomass under C3–C4 vegetation change: consideration of 13C fractionation and preferential substrate utilization. Soil Biol. Biochem. 43, 159–166. 10.1016/j.soilbio.2010.09.028

[B13] Blagodatsky S. A. Heinemeyer O. Richter J. (2000). Estimating the active and total soil microbial biomass by kinetic respiration analysis. Biol. Fertil. Soils 32, 73–81. 10.1007/s003740000219

[B14] Blume E. Bischoff M. Reichert J. Moorman T. Konopka A. Turco R. (2002). Surface and subsurface microbial biomass, community structure and metabolic activity as a function of soil depth and season. Appl. Soil Ecol. 20, 171–181. 10.1016/S0929-1393(02)00025-2

[B15] Bonkowski M. Cheng W. Griffiths B. S. Alphei J. Scheu S. (2000). Microbial-faunal interactions in the rhizosphere and effects on plant growth. Eur. J. Soil Biol. 36, 135–147. 10.1016/S1164-5563(00)01059-1

[B16] Buchkowski R. W. Schmitz O. J. Bradford M. A. (2015). Microbial stoichiometry overrides biomass as a regulator of soil carbon and nitrogen cycling. Ecology 96, 1139–1149. 10.1890/14-1327.1 26230033

[B17] Butterly C. Bünemann E. McNeill A. M. Baldock J. A. Marschner P. (2009). Carbon pulses but not phosphorus pulses are related to decreases in microbial biomass during repeated drying and rewetting of soils. Soil Biol. Biochem. 41, 1406–1416. 10.1016/j.soilbio.2009.03.01812027183

[B18] Devi N. B. Yadava P. (2006). Seasonal dynamics in soil microbial biomass C, N and P in a mixed-oak forest ecosystem of Manipur, North-east India. Appl. Soil Ecol. 31, 220–227. 10.1016/j.apsoil.2005.05.005

[B19] Dworkin J. Shah I. M. (2010). Exit from dormancy in microbial organisms. Nat. Rev. Microbiol. 8, 890–896. 10.1038/nrmicro2453 20972452

[B20] Fierer N. Schimel J. P. (2002). Effects of drying–rewetting frequency on soil carbon and nitrogen transformations. Soil Biol. Biochem. 34, 777–787. 10.1016/S0038-0717(02)00007-X

[B21] Frey S. D. Lee J. Melillo J. M. Six J. (2013). The temperature response of soil microbial efficiency and its feedback to climate. Nat. Clim. Change 3, 395–398. 10.1038/nclimate1796

[B22] Fujita Y. Witte J. P. M. Bodegom P. M. (2014). Incorporating microbial ecology concepts into global soil mineralization models to improve predictions of carbon and nitrogen fluxes. Global Biogeochem. Cycles 28, 223–238. 10.1002/2013GB004595

[B23] Geisen S. Bandow C. Römbke J. Bonkowski M. (2014). Soil water availability strongly alters the community composition of soil protists. Pedobiologia 57, 205–213. 10.1016/j.pedobi.2014.10.001

[B24] Goldberg D. (2015). Texas A&M University Geoservices. Available online at: http://geoservices.tamu.edu (Last accessed February 22, 2015).

[B25] Grogan P. Chapin F. (1999). Arctic soil respiration: effects of climate and vegetation depend on season. Ecosystems 2, 451–459. 10.1007/s100219900093

[B26] Gunapala N. Scow K. (1998). Dynamics of soil microbial biomass and activity in conventional and organic farming systems. Soil Biol. Biochem. 30, 805–816. 10.1016/S0038-0717(97)00162-4

[B27] Hagerty S. B. van Groenigen K. J. Allison S. D. Hungate B. A. Schwartz E. Koch G. W. (2014). Accelerated microbial turnover but constant growth efficiency with warming in soil. Nat. Clim. Change 4, 903–906. 10.1038/nclimate2361

[B28] He Y. Yang J. Zhuang Q. Harden J. W. McGuire A. D. Liu Y. (2015). Incorporating microbial dormancy dynamics into soil decomposition models to improve quantification of soil carbon dynamics of northern temperate forests. J. Geophys. Res. Biogeosci. 120, 2596–2611. 10.1002/2015JG003130

[B29] Holmes W. E. Zak D. R. (1994). Soil microbial biomass dynamics and net nitrogen mineralization in northern hardwood ecosystems. Soil Sci. Soc. Am. J. 58, 238–243. 10.2136/sssaj1994.03615995005800010036x

[B30] ICLIMATE (2015). Indiana State Climate Office. Available online at: http://iclimate.org/index.asp (Accessed February 22, 2015).

[B31] Ilstedt U. Nordgren A. Malmer A. (2000). Optimum soil water for soil respiration before and after amendment with glucose in humid tropical acrisols and a boreal mor layer. Soil Biol. Biochem. 32, 1591–1599. 10.1016/S0038-0717(00)00073-0

[B32] Jones S. E. Lennon J. T. (2010). Dormancy contributes to the maintenance of microbial diversity. Proc. Natl. Acad. Sci. U.S.A. 107, 5881–5886. 10.1073/pnas.0912765107 20231463 PMC2851880

[B33] Keidel L. Kammann C. Grünhage L. Moser G. Müller C. (2014). Positive feedback of elevated CO2 on soil respiration in late autumn and winter. Biogeosci. Discuss. 11, 8749–8787. 10.5194/bgd-11-8749-2014

[B34] Khomutova T. Demkina T. Demkin V. (2004). Estimation of the total and active microbial biomasses in buried subkurgan paleosoils of different age. Microbiology 73, 196–201. 10.1023/B:MICI.0000023989.04745.7b 15198037

[B35] Konopka A. (1999). Theoretical analysis of the starvation response under substrate pulses. Microb. Ecol. 38, 321–329. 10.1007/s002489900178 10758179

[B36] Lennon J. T. Jones S. E. (2011). Microbial seed banks: the ecological and evolutionary implications of dormancy. Nat. Rev. Microbiol. 9, 119–130. 10.1038/nrmicro2504 21233850

[B37] Li H.-J. Yan J.-X. Yue X.-F. Wang M.-B. (2008). Significance of soil temperature and moisture for soil respiration in a Chinese mountain area. Agric. Forest Meteorol. 148, 490–503. 10.1016/j.agrformet.2007.10.009

[B38] Manzoni S. Moyano F. Kätterer T. Schimel J. (2016). Modeling coupled enzymatic and solute transport controls on decomposition in drying soils. Soil Biol. Biochem. 95, 275–287. 10.1016/j.soilbio.2016.01.006

[B39] Manzoni S. Schaeffer S. Katul G. Porporato A. Schimel J. (2014). A theoretical analysis of microbial eco-physiological and diffusion limitations to carbon cycling in drying soils. Soil Biol. Biochem. 73, 69–83. 10.1016/j.soilbio.2014.02.008

[B40] Panikov N. S. (1995). Microbial Growth Kinetics. Springer Science & Business Media.

[B41] Panikov N. S. Sizova M. V. (1996). A kinetic method for estimating the biomass of microbial functional groups in soil. J. Microbiol. Methods 24, 219–230. 10.1016/0167-7012(95)00074-7

[B42] Payne W. J. (1970). Energy yields and growth of heterotrophs. Annu. Rev. Microbiol. 24, 17–52. 10.1146/annurev.mi.24.100170.000313 4927132

[B43] Pietikåinen J. Pettersson M. Bååth E. (2005). Comparison of temperature effects on soil respiration and bacterial and fungal growth rates. FEMS Microbiol. Ecol. 52, 49–58. 10.1016/j.femsec.2004.10.002 16329892

[B44] Pirt S. (1982). Maintenance energy: a general model for energy-limited and energy-sufficient growth. Arch. Microbiol. 133, 300–302. 10.1007/BF00521294 7171288

[B45] Placella S. A. Brodie E. L. Firestone M. K. (2012). Rainfall-induced carbon dioxide pulses result from sequential resuscitation of phylogenetically clustered microbial groups. Proc. Natl. Acad. Sci. U.S.A. 109, 10931–10936. 10.1073/pnas.1204306109 22715291 PMC3390866

[B46] Pomeroy L. R. (1974). The ocean's food web, a changing paradigm. Bioscience 24, 499–504. 10.2307/1296885

[B47] Reynolds L. L. Johnson B. R. Pfeifer-Meister L. Bridgham S. D. (2014). Soil respiration response to climate change in Pacific Northwest prairies is mediated by a regional Mediterranean climate gradient. Glob. Change Biol. 21, 487–500. 10.1111/gcb.12732 25205511

[B48] Rousk J. Bååth E. (2007). Fungal and bacterial growth in soil with plant materials of different C/N ratios. FEMS Microbiol. Ecol. 62, 258–267. 10.1111/j.1574-6941.2007.00398.x 17991019

[B49] Serna-Chavez H. M. Fierer N. Bodegom P. M. (2013). Global drivers and patterns of microbial abundance in soil. Glob. Ecol. Biogeogr. 22, 1162–1172. 10.1111/geb.12070

[B50] Speratti A. B. Whalen J. K. (2008). Carbon dioxide and nitrous oxide fluxes from soil as influenced by anecic and endogeic earthworms. Appl. Soil Ecol. 38, 27–33. 10.1016/j.apsoil.2007.08.009

[B51] Steinweg J. M. Dukes J. S. Wallenstein M. D. (2012). Modeling the effects of temperature and moisture on soil enzyme activity: linking laboratory assays to continuous field data. Soil Biol. Biochem. 55, 85–92. 10.1016/j.soilbio.2012.06.015

[B52] Stolpovsky K. Martinez-Lavanchy P. Heipieper H. J. Van Cappellen P. Thullner M. (2011). Incorporating dormancy in dynamic microbial community models. Ecol. Model. 222, 3092–3102. 10.1016/j.ecolmodel.2011.07.006

[B53] Sulman B. N. Phillips R. P. Oishi A. C. Shevliakova E. Pacala S. W. (2014). Microbe-driven turnover offsets mineral-mediated storage of soil carbon under elevated CO2. Nat. Clim. Change 4, 1099–1102. 10.1038/nclimate2436

[B54] Suseela V. Conant R. T. Wallenstein M. D. Dukes J. S. (2012). Effects of soil moisture on the temperature sensitivity of heterotrophic respiration vary seasonally in an old-field climate change experiment. Glob. Change Biol. 18, 336–348. 10.1111/j.1365-2486.2011.02516.x

[B55] Suseela V. Dukes J. S. (2013). The responses of soil and rhizosphere respiration to simulated climatic changes vary by season. Ecology 94, 403–413. 10.1890/12-0150.1 23691659

[B56] Tang J. Riley W. J. (2015). Weaker soil carbon-climate feedbacks resulting from microbial and abiotic interactions. Nat. Clim. Change 5, 56–60. 10.1038/nclimate2438

[B57] Tian J. Pausch J. Yu G. Blagodatskaya E. Gao Y. Kuzyakov Y. (2015). Aggregate size and their disruption affect ^14^C-labeled glucose mineralization and priming effect. Appl. Soil. Ecol. 90, 1–10.

[B58] USDA (2014). Soil Survey Staff, Natural Resources Conservation Service, United States Department of Agriculture. Web Soil Survey. Available online at: http://websoilsurvey.nrcs.usda.gov/ (Accessed September 24, 2014).

[B59] Van de Werf H. Verstraete W. (1987). Estimation of active soil microbial biomass by mathematical analysis of respiration curves: calibration of the test procedure. Soil Biol. Biochem. 19, 261–265. 10.1016/0038-0717(87)90007-1

[B60] Wang G. Jagadamma S. Mayes M. A. Schadt C. W. Steinweg J. M. Gu L.. (2015). Microbial dormancy improves development and experimental validation of ecosystem model. ISME J. 9, 226–237. 10.1038/ismej.2014.120 25012899 PMC4274429

[B61] Wang G. Mayes M. A. Gu L. Schadt C. W. (2014). Representation of dormant and active microbial dynamics for ecosystem modeling. PLoS One 9:e89252. 10.1371/journal.pone.0089252 24558490 PMC3928434

[B62] Wang Y. Liu H. Chung H. Yu L. Mi Z. Geng Y. (2014). Non-growing-season soil respiration is controlled by freezing and thawing processes in the summer-monsoon-dominated Tibetan alpine grassland. Glob. Biogeochem. Cycles 28, 1081–1095. 10.1002/2013GB004760

[B63] Wieder W. R. Allison S. D. Davidson E. A. Georgiou K. Hararuk O. He Y. (2015). Explicitly representing soil microbial processes in Earth system models. Glob. Biogeochem. Cycles 29, 1782–1800. 10.1002/2015GB005188

[B64] Wieder W. R. Bonan G. B. Allison S. D. (2013). Global soil carbon projections are improved by modelling microbial processes. Nat. Clim. Change 3, 909–912. 10.1038/nclimate1951

[B65] Wutzler T. Blagodatsky S. A. Blagodatskaya E. Kuzyakov Y. (2012). Soil microbial biomass and its activity estimated by kinetic respiration analysis–Statistical guidelines. Soil Biol. Biochem. 45, 102–112. 10.1016/j.soilbio.2011.10.004

[B66] Xu L. Baldocchi D. D. Tang J. (2004). How soil moisture, rain pulses, and growth alter the response of ecosystem respiration to temperature. Glob. Biogeochem. Cycles 18:GB4002 10.1029/2004GB002281

[B67] Xu X. Thornton P. E. Post W. M. (2013). A global analysis of soil microbial biomass carbon, nitrogen and phosphorus in terrestrial ecosystems. Glob. Ecol. Biogeogr. 22, 737–749. 10.1111/geb.12029

[B68] Yakushev A. (2015). Integral structural-functional method for characterizing microbial populations. Eurasian Soil Sci. 48, 378–394. 10.1134/S1064229315040110

[B69] Yu X. Zha T. Pang Z. Wu B. Wang X. Chen G.. (2011). Response of soil respiration to soil temperature and moisture in a 50-year-old oriental arborvitae plantation in China. PLoS ONE 6:e28397. 10.1371/journal.pone.0028397 22163012 PMC3232204

[B70] Zhang X. Niu G. Y. Elshall A. S. Ye M. Barron-Gafford G. A. Pavao-Zuckerman M. (2014). Assessing five evolving microbial enzyme models against field measurements from a semiarid savannah—What are the mechanisms of soil respiration pulses? Geophys. Res. Lett. 41, 6428–6434. 10.1002/2014GL061399

[B71] Zwietering M. Jongenburger I. Rombouts F. Van't Riet K. (1990). Modeling of the bacterial growth curve. Appl. Environ. Microbiol. 56, 1875–1881. 16348228 10.1128/aem.56.6.1875-1881.1990 PMC184525

