# Supplementary material for: Changes in the Size of the Active Microbial Pool Explain Short-Term Soil Respiratory Responses to Temperature and Moisture
Source: Front Microbiol. 2016 Apr 19;7:524. doi: 10.3389/fmicb.2016.00524 (PMC4836035; doi:10.3389/fmicb.2016.00524)
Supplement: Supplementary file 1 [file Table1.DOCX]

**Supplementary Table 1.** **Two-way ANOVA for SBR.** We calculated the percentage of the variance explained by each independent factor as the sum of squares for each independent factor divided by the total sum of squares. For example, in this case the sum of squares for temperature is 0.02970 and the total sum of squares is 0.10402, so the percentage of the SBR variance explained by temperature is 0.02970 /0.10402=0.286 or 28.6%.

|  | Df | Sum Sq | Mean Sq | F-value | P-value |
| --- | --- | --- | --- | --- | --- |
| Temp | 1 | 0.02970 | 0.02970 | 7.332 | 0.027 * |
| SM | 1 | 0.04048 | 0.04048 | 9.994 | 0.013 * |
| Temp:SM | 1 | 0.00143 | 0.00143 | 0.353 | 0.569 |
| Residuals | 8 | 0.03241 | 0.00405 |  |  |
| Total |  | 0.10402 |  |  |  |
