# Supplementary material for: Changes in the Size of the Active Microbial Pool Explain Short-Term Soil Respiratory Responses to Temperature and Moisture
Source: Front Microbiol. 2016 Apr 19;7:524. doi: 10.3389/fmicb.2016.00524 (PMC4836035; doi:10.3389/fmicb.2016.00524)
Supplement: Supplementary file 2 [file Table2.DOCX]

**Supplementary Table 2**. **Pairwise comparisons for SBR** using the Tukey’s HSD test with a confidence interval of 95%. Codes (here and elsewhere): ‘*’P < 0.05

| Treatments | 95% confidence interval | | P-value |
| --- | --- | --- | --- |
|  | **Lower limit** | **Upper limit** |  |
| heated-dry vs. unheated-dry | -0.089 | 0.244 | 0.483 |
| unheated-wet vs. unheated-dry | -0.072 | 0.261 | 0.333 |
| heated-wet vs. unheated-dry | 0.049 | 0.382 | 0.014* |
| unheated-wet vs. heated-dry | -0.150 | 0.183 | 0.988 |
| heated-wet vs. heated-dry | -0.028 | 0.304 | 0.108 |
| heated-wet vs. unheated-wet | -0.045 | 0.288 | 0.169 |
