# Supplementary material for: Changes in the Size of the Active Microbial Pool Explain Short-Term Soil Respiratory Responses to Temperature and Moisture
Source: Front Microbiol. 2016 Apr 19;7:524. doi: 10.3389/fmicb.2016.00524 (PMC4836035; doi:10.3389/fmicb.2016.00524)
Supplement: Supplementary file 3 [file Table3.DOCX]

**Supplementary Table S3**. **Two-way ANOVA for TMB.**

|  | Df | Sum Sq | Mean Sq | F-value | P-value |
| --- | --- | --- | --- | --- | --- |
| Temp | 1 | 3.2 | 3.2 | 0.037 | 0.853 |
| SM | 1 | 216.9 | 216.9 | 2.496 | 0.153 |
| Temp:SM | 1 | 815.5 | 815.5 | 9.387 | 0.016 * |
| Residuals | 8 | 695.0 | 86.9 |  |  |
| Total |  | 1730.6 |  |  |  |
