# Supplementary material for: Changes in the Size of the Active Microbial Pool Explain Short-Term Soil Respiratory Responses to Temperature and Moisture
Source: Front Microbiol. 2016 Apr 19;7:524. doi: 10.3389/fmicb.2016.00524 (PMC4836035; doi:10.3389/fmicb.2016.00524)
Supplement: Supplementary file 4 [file Table4.DOCX]

**Supplementary Table 4**. **Pairwise comparisons for TMB** using the Tukey’s HSD test with a confidence interval of 95%.

| Treatments | 95% confidence interval | | P-value |
| --- | --- | --- | --- |
|  | **Lower limit** | **Upper limit** |  |
| heated-dry vs. unheated-dry | -6.852 | 41.891 | 0.177 |
| unheated-wet vs. unheated-dry | -16.386 | 32.357 | 0.727 |
| heated-wet vs. unheated-dry | -31.842 | 16.901 | 0.764 |
| unheated-wet vs. heated-dry | -33.905 | 14.837 | 0.614 |
| heated-wet vs. heated-dry | -49.361 | -0.619 | 0.045* |
| heated-wet vs. unheated-wet | -39.827 | 8.915 | 0.254 |
