# Supplementary material for: Changes in the Size of the Active Microbial Pool Explain Short-Term Soil Respiratory Responses to Temperature and Moisture
Source: Front Microbiol. 2016 Apr 19;7:524. doi: 10.3389/fmicb.2016.00524 (PMC4836035; doi:10.3389/fmicb.2016.00524)
Supplement: Supplementary file 5 [file Table5.DOCX]

**Supplementary Table 5**. **Two-way ANOVA for log10-AMB.** Codes (here and elsewhere): ‘***’P< 0.001

|  | Df | Sum Sq | Mean Sq | F-value | P-value |
| --- | --- | --- | --- | --- | --- |
| Temp | 1 | 0.5436 | 0.5436 | 26.055 | 9.25e-04 *** |
| SM | 1 | 0.0657 | 0.0657 | 3.150 | 0.114 |
| Temp:SM | 1 | 0.1421 | 0.1421 | 6.813 | 0.031 * |
| Residuals | 8 | 0.1669 | 0.0209 |  |  |
| Total |  | 0.9183 |  |  |  |
