# Supplementary material for: Changes in the Size of the Active Microbial Pool Explain Short-Term Soil Respiratory Responses to Temperature and Moisture
Source: Front Microbiol. 2016 Apr 19;7:524. doi: 10.3389/fmicb.2016.00524 (PMC4836035; doi:10.3389/fmicb.2016.00524)
Supplement: Supplementary file 6 [file Table6.DOCX]

**Supplementary Table 6**. **Pairwise comparisons for log10-AMB** using the Tukey’s HSD test with a confidence interval of 95%. Codes (here and elsewhere): ‘**’P< 0.01, ‘†’ P<0.1

| Treatments | 95% confidence interval | | P-value |
| --- | --- | --- | --- |
|  | **Lower limit** | **Upper limit** |  |
| heated-dry vs. unheated-dry | -0.170 | 0.586 | 0.355 |
| unheated-wet vs. unheated-dry | -0.447 | 0.308 | 0.932 |
| heated-wet vs. unheated-dry | 0.196 | 0.951 | 0.005** |
| unheated-wet vs. heated-dry | -0.655 | 0.100 | 0.164 |
| heated-wet vs. heated-dry | -0.012 | 0.743 | 0.058 † |
| heated-wet vs. unheated-wet | 0.266 | 1.021 | 0.003** |
