# Supplementary material for: Changes in the Size of the Active Microbial Pool Explain Short-Term Soil Respiratory Responses to Temperature and Moisture
Source: Front Microbiol. 2016 Apr 19;7:524. doi: 10.3389/fmicb.2016.00524 (PMC4836035; doi:10.3389/fmicb.2016.00524)
Supplement: Supplementary file 7 [file Table7.DOCX]

**Supplementary Table 7**. **Two-way ANOVA for *t_lag._***

|  | Df | Sum Sq | Mean Sq | F-value | P-value |
| --- | --- | --- | --- | --- | --- |
| Temp | 1 | 219.94 | 219.94 | 86.592 | 1.45e-05 ** |
| SM | 1 | 16.07 | 16.07 | 6.328 | 0.036 * |
| Temp:SM | 1 | 20.59 | 20.59 | 8.108 | 0.022 * |
| Residuals | 8 | 20.32 | 2.54 |  |  |
| Total |  | 276.92 |  |  |  |
