# Supplementary material for: Changes in the Size of the Active Microbial Pool Explain Short-Term Soil Respiratory Responses to Temperature and Moisture
Source: Front Microbiol. 2016 Apr 19;7:524. doi: 10.3389/fmicb.2016.00524 (PMC4836035; doi:10.3389/fmicb.2016.00524)
Supplement: Supplementary file 8 [file Table8.DOCX]

**Supplementary Table 8**. **Pairwise comparisons for *t_lag_*** using the Tukey’s HSD test with a confidence interval of 95%.

| Treatments | 95% confidence interval | | P-value |
| --- | --- | --- | --- |
|  | **Lower limit** | **Upper limit** |  |
| heated-dry vs. unheated-dry | -10.109 | -1.775 | 0.008** |
| unheated-wet vs. unheated-dry | -3.862 | 4.472 | 0.995 |
| heated-wet vs. unheated-dry | -15.044 | -6.710 | 1.47e-04** |
| unheated-wet vs. heated-dry | 2.081 | 10.415 | 0.006** |
| heated-wet vs. heated-dry | -9.102 | -0.768 | 0.022* |
| heated-wet vs. unheated-wet | -15.349 | -7.015 | 1.20e-04** |
