# Supplementary material for: Changes in the Size of the Active Microbial Pool Explain Short-Term Soil Respiratory Responses to Temperature and Moisture
Source: Front Microbiol. 2016 Apr 19;7:524. doi: 10.3389/fmicb.2016.00524 (PMC4836035; doi:10.3389/fmicb.2016.00524)
Supplement: Supplementary file 9 [file Table9.DOCX]

**Supplementary Table 9**. **Two-way ANOVA for *μ.***

|  | Df | Sum Sq | Mean Sq | F-value | P-value |
| --- | --- | --- | --- | --- | --- |
| Temp | 1 | 0.000325 | 0.000325 | 0.332 | 0.5804 |
| SM | 1 | 0.000141 | 0.000141 | 0.144 | 0.7143 |
| Temp:SM | 1 | 0.004069 | 0.004069 | 4.155 | 0.0759 † |
| Residuals | 8 | 0.007834 | 0.000979 |  |  |
| Total |  | 0.012369 |  |  |  |
