# Supplementary material for: Changes in the Size of the Active Microbial Pool Explain Short-Term Soil Respiratory Responses to Temperature and Moisture
Source: Front Microbiol. 2016 Apr 19;7:524. doi: 10.3389/fmicb.2016.00524 (PMC4836035; doi:10.3389/fmicb.2016.00524)
Supplement: Supplementary file 10 [file Table10.DOCX]

**Supplementary Table 10**. **Pairwise comparisons for *μ*** using the Tukey’s HSD test with a confidence interval of 95%.

| Treatments | 95% confidence interval | | P-value |
| --- | --- | --- | --- |
|  | **Lower limit** | **Upper limit** |  |
| heated-dry vs. unheated-dry | -0.03459 | 0.129059 | 0.319893 |
| unheated-wet vs. unheated-dry | -0.05185 | 0.111796 | 0.658718 |
| heated-wet vs. unheated-dry | -0.07827 | 0.085376 | 0.998957 |
| unheated-wet vs. heated-dry | -0.09909 | 0.064559 | 0.90344 |
| heated-wet vs. heated-dry | -0.12551 | 0.038139 | 0.378694 |
| heated-wet vs. unheated-wet | -0.10824 | 0.055403 | 0.735585 |
