# Supplementary material for: Changes in the Size of the Active Microbial Pool Explain Short-Term Soil Respiratory Responses to Temperature and Moisture
Source: Front Microbiol. 2016 Apr 19;7:524. doi: 10.3389/fmicb.2016.00524 (PMC4836035; doi:10.3389/fmicb.2016.00524)
Supplement: Supplementary file 11 [file Table11.DOCX]

**Supplementary Table 11**. **Soil basal respiration and microbial parameters** (estimated by kinetic respiration analysis) at different temperature and soil moisture conditions.

| T (°C) | SM (% SDW) | SBR | *R_u_* | *R_c_* | *μ* | TMB | r_0_ | AMB | *t_lag_* |
| --- | --- | --- | --- | --- | --- | --- | --- | --- | --- |
| 24 | **10** | 0.171 | 1.483 | 0.168 | 0.183 | 110.420 | 0.011 | 1.235 | 11.883 |
|  |  | 0.142 | 1.825 | 0.119 | 0.190 | 130.389 | 0.006 | 0.847 | 14.337 |
|  |  | 0.182 | 1.970 | 0.036 | 0.263 | 101.141 | 0.002 | 0.182 | 15.247 |
|  | **20** | 0.355 | 2.138 | 0.093 | 0.236 | 122.914 | 0.004 | 0.535 | 13.272 |
|  |  | 0.196 | 2.352 | 0.032 | 0.277 | 114.938 | 0.001 | 0.155 | 15.565 |
|  |  | 0.227 | 2.023 | 0.111 | 0.214 | 128.054 | 0.005 | 0.698 | 13.546 |
| 33 | **10** | 0.19 | 2.331 | 0.244 | 0.260 | 122.204 | 0.010 | 1.268 | 8.669 |
|  |  | 0.216 | 2.879 | 0.172 | 0.290 | 134.879 | 0.006 | 0.801 | 9.717 |
|  |  | 0.322 | 2.259 | 0.680 | 0.229 | 137.425 | 0.029 | 4.014 | 5.254 |
|  | **20** | 0.354 | 1.584 | 0.763 | 0.219 | 102.410 | 0.046 | 4.704 | 3.340 |
|  |  | 0.337 | 1.609 | 0.899 | 0.214 | 107.397 | 0.053 | 5.683 | 2.725 |
|  |  | 0.451 | 1.658 | 0.913 | 0.215 | 109.731 | 0.052 | 5.728 | 2.771 |
