# Supplementary figures and images for: Changes in the Size of the Active Microbial Pool Explain Short-Term Soil Respiratory Responses to Temperature and Moisture
Source: Front Microbiol. 2016 Apr 19;7:524. doi: 10.3389/fmicb.2016.00524 (PMC4836035; doi:10.3389/fmicb.2016.00524)

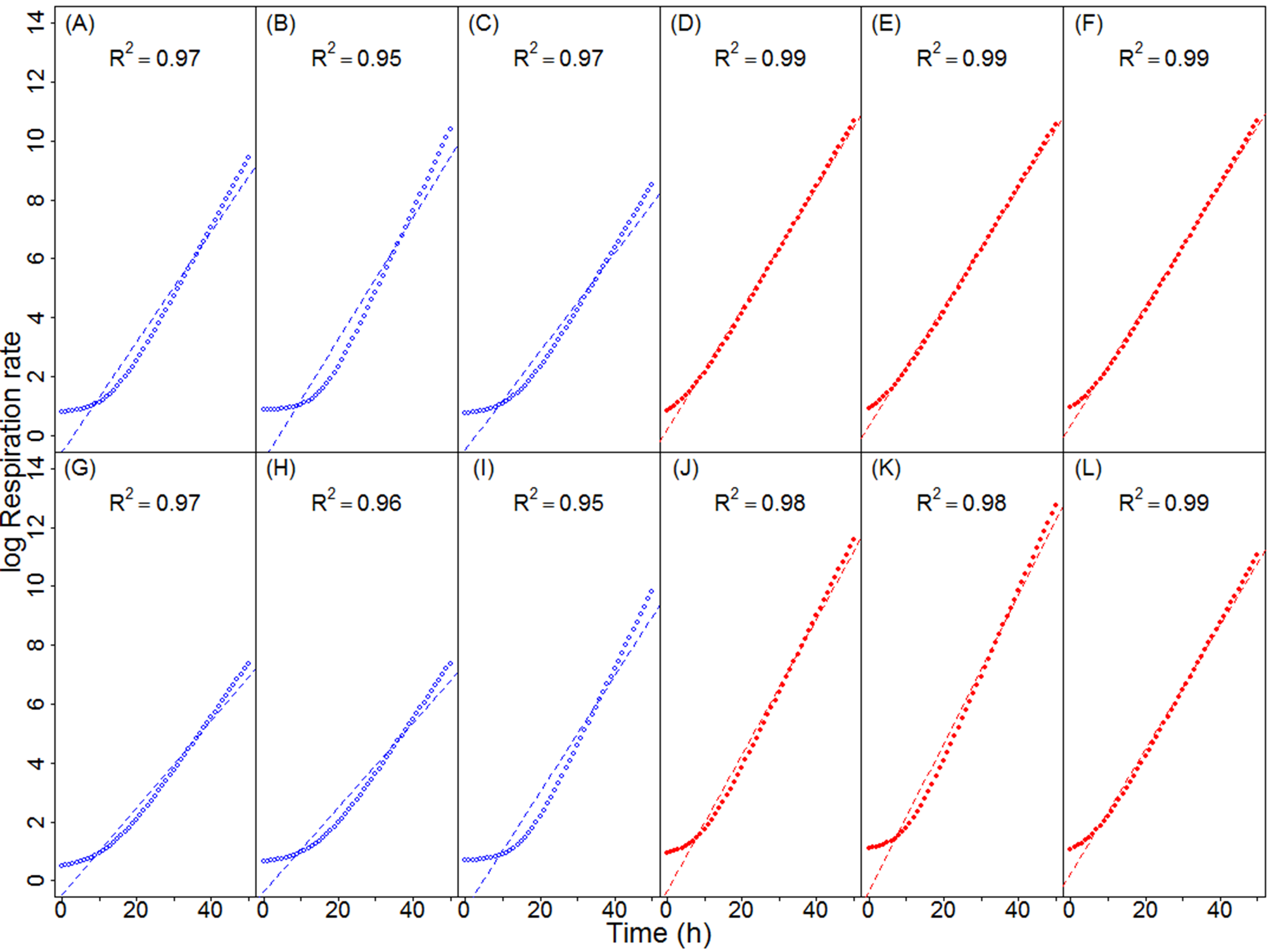

Supplement: Supplementary file 12 [file Image1.TIF]
